# Far-Infrared Emission Properties and Thermogravimetric Analysis of Ceramic-Embedded Polyurethane Films

**DOI:** 10.3390/polym13050686

**Published:** 2021-02-25

**Authors:** Ashik Md Faisal, Fabien Salaün, Stéphane Giraud, Ada Ferri, Yan Chen, Lichuan Wang

**Affiliations:** 1 Politecnico di Torino, DISAT, Corso Duca Degli Abruzzi 24, 10129 Turin, Italy; ada.ferri@polito.it; 2 University of Lille Nord de France, F-5900 Lille, France; fabien.salaun@ensait.fr (F.S.); stephane.giraud@ensait.fr (S.G.); 3 ENSAIT, GMTEX, F-59100 Roubaix, France; 4 College of Textile and Clothing Engineering, Soochow University, Suzhou 215123, China; yanchen@suda.edu.cn (Y.C.); lcwang@suda.edu.cn (L.W.)

**Keywords:** far-infrared ray, ceramic particles, polyurethane, emissivity, functional textiles

## Abstract

The far-infrared ray (FIR) is one kind of electromagnetic wave employed for numerous bio-interactive applications such as body thermoregulation, infrared therapy, etc. Tuning the FIR-emitting property of the functional textile surface can initiate a new horizon to utilize this property in sportswear or even smart textiles. Ceramic particles were studied for their unique ability to constantly emit FIR rays. The purpose of this research is to characterize the FIR emission properties and the thermogravimetric analysis of ceramic-embedded polyurethane films. For this purpose, ceramic particles such as aluminum oxide, silicon dioxide, and titanium dioxide were incorporated (individually) with water-based polyurethane (WPU) binder by a sonication technique to make a thin layer of film. Significant improvement in FIR emissive property of the films was found when using different ceramic particles into the polyurethane films. Reflection and transmission at the FIR range were measured with a gold integrating sphere by Fourier-transform infrared (FTIR) spectrometer. The samples were also characterized by thermogravimetric analysis (TGA). Different physical tests, such as tensile strength and contact angle measurements, were performed to illustrate the mechanical properties of the films. The study suggested that the mechanical properties of the polyurethane films were significantly influenced by the addition of ceramic particles.

## 1. Introduction

Polyurethane (PU) has been effectively used as a coating material due to its unique properties of corrosion resistance, microbial resistance, and durability against wear and weathering [1,2]. Nowadays, PU binder gains a great deal of attention due to the potentiality in selection of monomeric materials, which allows for a wide range of applications [3,4]. Water-based polyurethane (WPU) binders are the composed of low or non-volatile organic compounds, and they are widely used as coating materials since they are environmentally friendly and non-toxic [5,6]. Unfortunately, PU coatings are often hygroscopic, which causes permeation of aggressive ions such as oxygen and chloride; long exposure in ultraviolet (UV) radiation causes photochemical degradation of PU, which makes the coating yellowish. Both of this process gradually causes PU disintegration which affects its mechanical and optical properties [7,8]. Several studies suggest that the addition of inorganic particles (such as cerium oxide, titania, and zinc oxide) may improve the chemical and mechanical resistance of the PU (hybrid) coatings [9,10,11,12,13].

Ceramic particles would be a great choice in order to improve the functionality as well as the chemical and mechanical resistance of the PU coatings. Ceramic particles, such as aluminum oxide, silicon dioxide, titanium dioxide, were studied for their unique property to constantly emit FIR rays [14]. The application of aluminum oxide could significantly increase the surface emissivity of coated composite material [15,16]. Silicon dioxide-based finishing shown excellent thermal insulation properties on cotton fabrics even though the emission properties of silicon dioxide were not so far deeply analyzed [17]. Ceramic coatings containing titanium dioxide showed a greater infrared emissivity value (about 90%) in the range of resonance wavelength [18,19]. Ceramic particles have also shown good antimicrobial properties when incorporated into textiles as a coating material [20]. Polymer-based ceramic composites were proposed for applications like nano-membranes and optical applications due to their FIR-emissive properties [21,22].

The utilization of FIR ray-associated textile products has shown interest from the sportswear industry [23] because of its potential bio-interactive application [24,25,26] and body thermoregulation [27,28,29,30]. In order to amplify the desired emissive properties of the engineered textile surface, researchers have successfully exhibited several methods, including the use of dyes and pigments, electro-spinning of different materials, insertion of inorganic materials, or even modification of the cross-sectional shape of the fiber [31]. 

FIR is one kind of invisible electromagnetic wave with the wavelength range from 0.75 μm to 1000 μm. FIR has been claimed for the generation of resonance to the human body and is also known as “thermal ray” [32]. The electromagnetic spectrum is divided into several wavelength regions (from 10^−5^ nm to 10^3^ m) by methods of detecting and producing radiation. All electromagnetic radiation is primarily the same and subordinate by the same laws, and the only difference is the wavelength. The absorption wavelength range of most organic compounds is from 6 μm to 14 μm, which is also known as the resonance wavelength (life rays) [33,34]. The human body absorption wavelength is around 10 μm and according to Wien’s law, the peak wavelength radiation (λ_peak_) of human skin is estimated to be 9.34 μm [35]. Resonance occurs when the elements closer to the human body emit or reflect a similar wavelength as the human body. It is reported that resonance enhances blood circulation, which eventually generates metabolic heat [32].

Therefore, the addition of FIR-emitting ceramic particles as a filler into the PU coating is a promising way to improve the functionality and the performance of the composites. In the work presented here, ceramic particles have been incorporated into the water-based polyurethane binder to utilize the FIR radiation properties of ceramics. The emissive properties (in FIR range) of thin-layer polyurethane films incorporated with different ceramic particles have been investigated. Enhancement of FIR emissive properties, especially at the peak wavelength region, was found due to the addition of ceramic particles into the PU composites. Thermogravimetric analysis (TG) and different physical tests were performed to characterize and illustrate the mechanical properties of the films. The study suggested that the addition of ceramic particles have a significant influence on the mechanical properties of the PU composite.

## 2. Materials and Methods 

In this study, three different ceramic particles were individually incorporated with a water-based polyurethane binder to independently investigate their FIR emissive properties. The WPU binder Lurapret N5657 liq (with 40% solid content) was supplied by Archroma (Reinach, Switzerland) and ceramic nanoparticles (aluminum oxide, silicon dioxide, titanium dioxide) were purchased from Evonik (Essen, Germany). The detailed specification of the samples are shown in Table 1.

**Figure 1:**
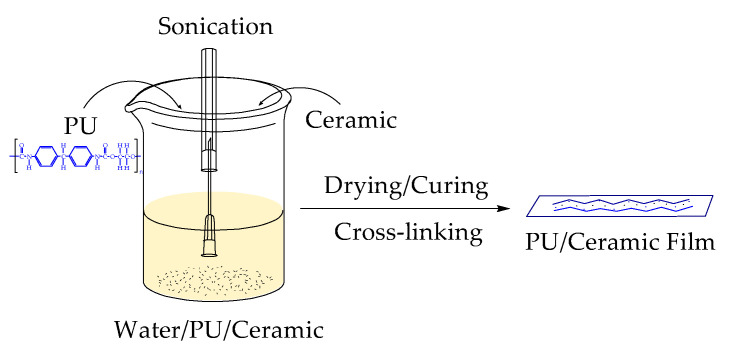
Sample preparation scheme for polyurethane composites with different ceramics.

Based on the solid content of the end product, 5 wt.% of ceramic particles (weight-percentage) were incorporated with a water-based polyurethane (PU) binder. The concentration of ceramic particles in water was kept at 10 g/L. Ceramic particles were initially dispersed to water using the probe-sonication technique for one hour and then the water-based polyurethane binder was slowly added to the mixture. For a uniform mixture of the solution, further probe-sonication and magnetic stirring were applied. Then, the mixture was put into a Teflon mold to make a thin layer of polyurethane film. The films were kept to the Teflon mold at room temperature (~20 °C) for 24 h and then dried in an incubator at 95 °C for 30 min. The films were then cured at 130 °C for 6 min to finish the crosslinking of the polyurethane. Crosslinking was preliminarily observed by the extent of swelling (solvent up-take). The thickness of each sample was measured around 0.6 mm. An illustration of the sample preparation technique can be seen in Figure 1.

A Fourier-transform infrared (FTIR) spectrometer from Pike Technologies (Fitchburg, WI, USA) with an integrated reflective gold sphere was used [1] to measure the spectral transmittance (τ) and reflectance (ρ) at the wavelength range from 6 μm to 14 μm in standard atmospheric conditions. The emissivity (ε) of the films were then calculated by Kirchhoff’s Law of thermal radiation: ε = 1 − τ − ρ [36]. The surface morphology of the samples was investigated using the LEO 435 VP scanning electron microscope (SEM) from Leica (Richmond, IL, USA). At first, the sample was electroplated and then kept onto the SEM specimen holder and photographed from above. A field emission scanning electron microscope (FESEM) was used to achieve well-defined images down to the micrometer range. Thermogravimetric analysis of the samples was carried out using a TGA 3+ (Mettler Toledo, Columbus, OH, USA). Thermogravimetric analysis was performed at the temperature range from 20 °C to 700 °C, at a heating rate of 10 °C per minute, at an inert atmosphere (inert nitrogen gas condition at a constant flow of 50 mL/min during the experiment). The contact angle of the samples for different liquids was measured by the DIGIDROP contact angle meter. Tensile properties were measured according to the ISO 527-1 standard method with a cell force of 1 kN and a drawing speed of 1 mm/min. The tests were performed at standard atmospheric conditions (a temperature of 20 ± 2 °C and relative humidity of 65 ± 5%).

## 3. Results and Discussion

### 3.1. SEM Analysis of Composite Films

**Figure 2:**
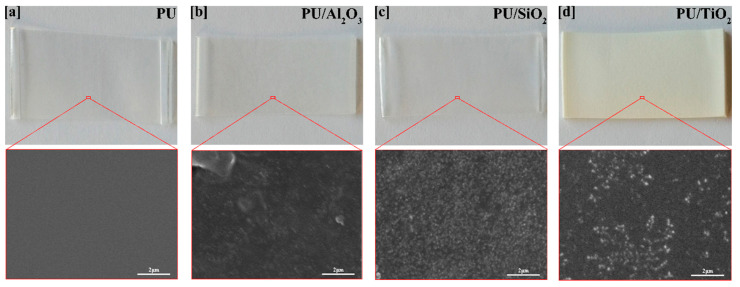
Photos of different samples and scanning electron microscope (SEM) images of the PU, PU/ceramic embedded films: (**a**) PU, (**b**) PU/Al_2_O_3_, (**c**) PU/SiO_2_, and (**d**) Pu/TiO_2._

The surface morphology of the samples was investigated via FESEM images and shown in Figure 2. By magnifying the samples at 9000 times and comparing Figure 2a (PU without ceramic particles) with Figure 2b–d, the presence of ceramic particles were clearly observed. Ceramic particles have the tendency to agglomerate while using water as a solvent due to the high polarity of water [37]. In this work, a sonication technique was used to disperse the ceramic particles uniformly and to reduce the agglomeration of the ceramic particles. According to the SEM observation, the ceramic particles were seen to be well dispersed in the polyurethane matrix. The mean observed aggregate size of the ceramics was roughly 100 nm to 500 nm. The presence of spherical particles of low diameters were documented by the SEM microphotograph, which formed larger agglomerates and aggregates.

### 3.2. FIR Emission Properties of Composite Films

**Figure 3:**
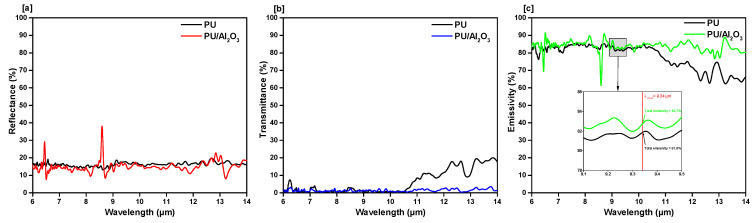
FIR property characterization of aluminum oxide incorporated PU films. Fourier-transform infrared (FTIR) measured (**a**) reflectance, (**b**) transmittance, (**c**) emissivity.

**Figure 4:**
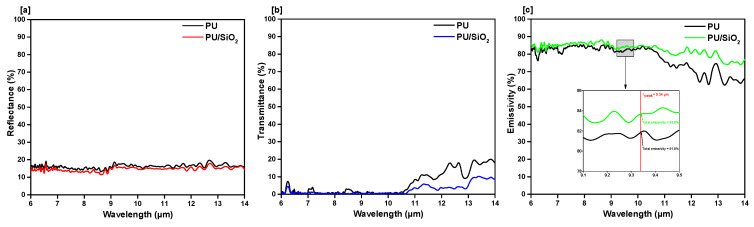
FIR property characterization of silicon dioxide incorporated PU films. FTIR measured (**a**) reflectance, (**b**) transmittance, (**c**) emissivity.

**Figure 5:**
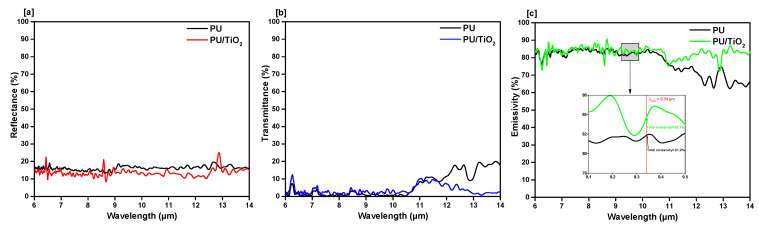
FIR property characterization of titanium dioxide incorporated PU films. FTIR measured (**a**) reflectance, (**b**) transmittance, (**c**) emissivity.

To make a qualitative analysis of the FIR emission properties between the PU films (with and without ceramic particles), FTIR spectra was performed at the wavelength range of 6 µm to 14 µm (resonance wavelength). The results of reflectance, transmittance, and emissivity of different samples can be seen in Figure 3, Figure 4 and Figure 5. It was observed that the overall emissivity of the ceramics embedded PU films were significantly higher than the ordinary (films without ceramic) ones. At the peak wavelength (λ_peak_ = 9.34 µm) of human body radiation, the emissivity of the aluminum oxide (Al_2_O_3_) incorporated film was found to be 82.7%, which was higher than the emissivity of the common polyurethane film (81.8%, shown in Figure 3c). At the mid-IR spectral range, from 10.5 µm to 14 µm, the influence of added ceramic particles on the emissivity was even greater.

At the peak wavelength of human body radiation, the emissivity of the silicon dioxide (SiO_2_) incorporated film was found 83.6% (shown in Figure 4c), which is higher than the ordinary PU film. The addition of silicon dioxide to the PU film provided good emissive properties, especially at the mid-IR spectral range. 

The emissivity at the peak wavelength of the titanium dioxide (TiO_2_)-added PU film was found 83.7% (shown in Figure 5c), which is also higher than the emissivity of the common PU film (81.8%).

The addition of ceramic particles into PU composite offers good emissive properties, especially at the mid-IR spectral range. The emissivity of the titanium dioxide added PU film was shown better results compared to other ceramic particles added PU films. Therefore, the addition of ceramic particles improved the FIR emissive properties of the PU composite films. These films can be used as an FIR emissive coating which is similar to currently available FIR-emitting materials [4,38].

### 3.3. TG Properties of Composite Films

**Figure 6:**
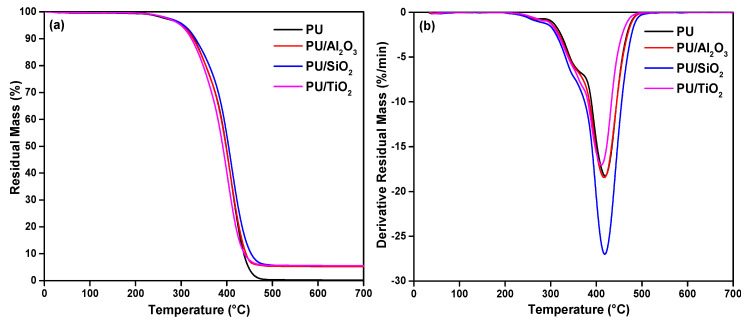
Thermogravimetric (TG) analysis of different PU/ceramic composites; (**a**): residual mass (%) and (**b**) derivative residual mass (%/min).

The effect of ceramic particles on the thermal stability of the polyurethane films was measured using thermogravimetric analysis (carried out in inert conditions). The results of residual mass and the derivative residual mass are shown in Figure 6. The thermal behavior of both PU and PU/ceramics displayed multi-step degradation. The main step of degradation of microcapsules containing ceramic particles occurred between 368 °C and 408 °C. The decomposition of PU and PU/ceramics is characterized by the onset temperature at 5% weight loss (T_onset5%_), starting at around 316 °C.

The main decomposition of PU corresponding to PU/ceramics took place at 420 °C. Thermogravimetric data of different films are shown in Table 2. There is almost no char residue remaining in the PU sample at over 480 °C. The weight loss of PU/ceramic (PU/Al_2_O_3_: 315°C, PU/SiO_2_: 318 °C, and PU/TiO_2_: 312 °C) composites began around the same temperature of PU (T_onset5%_: 316 °C). This suggests that the thermal stability of polyurethane films is not adversely affected by the the incorporation of ceramic particles. However, the thermal degradation process should be different as the ceramic particles have an effect on the structure of PU composite [39]. Around 5% of char residue was found for PU/ceramic films, while for PU film, the residue was 0.2%. This indicates that the addition of ceramic particles increases the residual mass of the PU composites. It was also observed that the content of the ceramic particle and the method of sample preparation had minimal effect on T_max_ and T_onset_.

### 3.4. Mechanical Properties of Composite Films

The mechanical properties of polymer composites can be influenced by the reinforcing effects of a kind of filler added to the polymer [40]. Properties of fillers, such as the particle size, aggregate size, degree of dispersion, shape, surface characteristics, and aspect ratio can influence the mechanical properties of polymer composites. The effect of ceramic particles on the tensile properties of the polyurethane films are listed in Table 3. The addition of ceramic particles can affect the crystallization of the polyurethane matrix. The ceramic particles can change the structural characteristics of the semi-crystalline matrix which can affect the tensile strength of the films [4]. The addition of ceramic particles resulted in the increment of tensile strength for the films which indicates the uniform dispersion of ceramic particles into the films. The elongation at break of the PU/ceramic composites decreased significantly compared to the ordinary PU film. The addition of ceramic particles into the PU composite seems to cause the failure of the films at low elongation, most likely due to the shape, surface, and brittle fracture as seen in Figure 2.

### 3.5. Contact Angle Analysis of Composite Films

The comparison of contact angles for different measuring liquids of the samples can reveal the changes in the hydrophilic nature of the films, as the polyurethane was incorporated with different ceramic particles. The contact angle of the samples for different measuring liquids is shown in Table 4. The contact angle of a solid surface may vary due to several factors, such as the wettability of the surface, nature of the material (hydrophilic or hydrophobic), and roughness of the surface [41]. From observing the samples, it was found that the contact angles for different measuring liquids increased with the addition of ceramic particles into the PU composites; which revealed that the harshness developed by the ceramic particle increases the hydrophobicity of the PU composites.

## 4. Conclusions

The aim of this work was to analyze the FIR emissive properties of ceramic-embedded PU films and the effect of ceramic particles on the mechanical properties of PU films. The results showed that the addition of ceramic particles has a significant influence on the FIR emissive properties of the polyurethane films. The reinforcing effect of ceramic particles also influenced the mechanical properties of the polyurethane films. This work will provide useful guidance towards the implementation of the ceramic particle as an FIR material to composites.

## Figures and Tables

**Table 1:** Compositions and characteristics of the samples.

Sample ID	Name of Ceramic	Ceramic Type	Particle Size of Ceramic (nm)	Ceramic Content (wt. %)	Thickness of Film (mm)
PU	-	-	-	0	0.6 ± 0.02
PU/Al_2_O_3_	Aluminum oxide	Hydrophobic	7~20	5	0.6 ± 0.04
PU/SiO_2_	Silicon dioxide	Hydrophobic	5~50	5	0.6 ± 0.03
PU/TiO_2_	Titanium dioxide	Hydrophilic	7~100	5	0.6 ± 0.03

**Table 2:** Thermogravimetric data of different PU/ceramic composites.

Sample ID	T_onset5%_ (°C)	Mass Losses for Each Step						Residue at 600 °C (%)
		Step I		Step II		Step III		
		Temperature Range (°C)	Mass Losses (%)	Temperature Range (°C)	Mass Losses (%)	Temperature Range (°C)	Mass Losses (%)	
PU	316	261–270	2.5	368–376	28.6	416–425	68.1	0.2
PU/Al_2_O_3_	316	261–270	2.3	351–360	20.8	416–425	71.2	5.3
PU/SiO_2_	319	278–286	3.0	351–360	17.8	416–425	73.2	5.6
PU/TiO_2_	313	278–286	3.3	368–376	33.4	408–416	57.3	5.6

**Table 3:** Mechanical properties of different PU/ceramic composite films.

Tensile Properties	PU	PU/Al_2_O_3_	PU/SiO_2_	PU/TiO_2_
Tensile strength (MPa)	18 ± 0.2	21 ± 0.4	19 ± 0.3	20 ± 0.8
Elongation at break (%)	439 ± 1.9	233 ± 0.8	225 ± 0.5	228 ± 0.5

**Table 4:** The contact angle of the samples for different measuring liquids.

Sample ID	Average Contact Angle (°)	
	Diiodomethane	Water
PU	40 ± 0.2	44 ± 0.3
PU/Al_2_O_3_	50 ± 0.3	60 ± 0.4
PU/SiO_2_	49 ± 0.3	54 ± 0.3
PU/TiO_2_	50 ± 0.3	54 ± 0.2

## Data Availability

The data presented in this study are available on request from the corresponding author.

## References

[1] Tielemans M., Bleus J.-P. New radiation-curable polyurethane dispersions for outdoor application on wood. Proceedings of the 5th International Woodcoatings Congress.

[2] Saha S., Kocaefe D., Boluk Y., Pichette A. (2013). Surface degradation of CeO_2_ stabilized acrylic polyurethane coated thermally treated jack pine during accelerated weathering. Appl. Surf. Sci..

[3] Patti A., Acierno D. (2019). The effect of silica/polyurethane waterborne dispersion on the perforating features of impregnated polypropylene-based fabric. Text. Res. J..

[4] Xiong Y., Zou Y., Cai S., Liu H., Huang S., Li H. (2019). Processing and characterization of polymer-based far-infrared composite materials. Polymers.

[5] Qiu F., Xu H., Wang Y., Xu J., Yang D. (2012). Preparation, characterization and properties of UV-curable waterborne polyurethane acrylate/SiO_2_ coating. J. Coatings Technol. Res..

[6] Caminero M.Á., Chacón J.M., García-Plaza E., Núñez P.J., Reverte J.M., Becar J.P. (2019). Additive manufacturing of PLA-based composites using fused filament fabrication: Effect of graphene nanoplatelet reinforcement on mechanical properties, dimensional accuracy and texture. Polymers.

[7] Heidarian M., Shishesaz M.R., Kassiriha S.M., Nematollahi M. (2010). Characterization of structure and corrosion resistivity of polyurethane/organoclay nanocomposite coatings prepared through an ultrasonication assisted process. Prog. Org. Coat..

[8] Rosu D., Rosu L., Cascaval C.N. (2009). IR-change and yellowing of polyurethane as a result of UV irradiation. Polym. Degrad. Stab..

[9] Saha S., Kocaefe D., Krause C., Larouche T. (2011). Effect of Titania and zinc oxide particles on acrylic polyurethane coating performance. Prog. Org. Coat..

[10] Figueira R.B., Silva C.J.R., Pereira E.V. (2015). Organic–inorganic hybrid sol–gel coatings for metal corrosion protection: A review of recent progress. J. Coat. Technol. Res..

[11] Wang X., Hu J., Li Y., Zhang J., Ding Y. (2015). The surface properties and corrosion resistance of fluorinated polyurethane coatings. J. Fluor. Chem..

[12] Bautista Y., Gomez M., Ribes C., Sanz V. (2011). Relation between the scratch resistance and the chemical structure of organic–inorganic hybrid coatings. Prog. Org. Coat..

[13] Saadat-Monfared A., Mohseni M., Tabatabaei M.H. (2012). Polyurethane nanocomposite films containing nano-cerium oxide as UV absorber. Part 1. Static and dynamic light scattering, small angle neutron scattering and optical studies. Colloids Surf. A Physicochem. Eng. Asp..

[14] Kubiliene D., Sankauskaite A., Abraitiene A., Krauledas S., Barauskas R. (2016). Investigation of thermal properties of ceramic-containing knitted textile materials. Fibres Text. East. Eur..

[15] Bartl J., Baranek M. (2004). Emissivity of aluminium and its importance for radiometric measurement. Meas. Sci. Rev..

[16] Abbas A., Zhao Y., Ali U., Lin T. (2017). Improving heat-retaining property of cotton fabrics through surface coatings. J. Text. Inst..

[17] Rosace G., Guido E., Colleoni C., Barigozzi G. (2016). Influence of textile structure and silica based finishing on thermal insulation properties of cotton fabrics. Int. J. Polym. Sci..

[18] Wang Z., Wang Y., Liu Y., Xu J., Guo L., Zhou Y., Ouyang J., Dai J. (2011). Microstructure and infrared emissivity property of coating containing TiO_2_ formed on titanium alloy by microarc oxidation. Curr. Appl. Phys..

[19] Hanada T., Aikawa T., Soga N. (1984). ChemInform abstract: Physical properties and structure of rf-sputtered amorphous films in the system titania-silica. Chem. Inf..

[20] Faisal A.M., Salaün F., Giraud S., Ferri A., Chen Y., Wang L. Analysis of the thermal comfort properties and FIR infrared emission characteristics of ceramic nanofillers imbedded fabrics. Proceedings of the 19th World Textile Conference-Autex.

[21] Wang H.-F. (2012). Bone and joint protection ability of ceramic material with biological effects. Chin. J. Physiol..

[22] Leung T.-K., Lin Y.-S., Lee C.-M., Chen Y.-C., Shang H.-F., Hsiao S.-Y., Chang H.-T., Chao J.-S. (2011). Direct and indirect effects of ceramic far infrared radiation on the hydrogen peroxide-scavenging capacity and on murine macrophages under oxidative stress. J. Med. Biol. Eng..

[23] Worobets J.T., Skolnik E.R., Stefanyshyn D.J. (2015). Apparel with far infrared radiation for decreasing an athlete’s oxygen consumption during submaximal exercise. Res. J. Text. Appar..

[24] Conrado L.A.L., Munin E. (2011). Reduction in body measurements after use of a garment made with synthetic fibers embedded with ceramic nanoparticles. J. Cosmet. Dermatol..

[25] Vigneshwaran N., Kumar S., Kathe A.A., Varadarajan P.V., Prasad V. (2006). Functional finishing of cotton fabrics using zinc oxide–soluble starch nanocomposites. Nanotechnology.

[26] Birol H., Rambo C.R., Guiotoku M., Hotza D. (2013). Preparation of ceramic nanoparticlesvia cellulose-assisted glycine nitrate process: A review. RSC Adv..

[27] Vatansever F., Hamblin M.R. (2012). Far infrared radiation (FIR): Its biological effects and medical applications. Photon. Lasers Med..

[28] Hsu P.-C., Song A.Y., Catrysse P.B., Liu C., Peng Y., Xie J., Fan S., Cui Y. (2016). Radiative human body cooling by nanoporous polyethylene textile. Science.

[29] Faisal A.M., Salaün F., Giraud S., Ferri A., Chen Y., Wang L. Characterization and thermographic analysis of fir emitting ceramic nanoparticle embedded films. Proceedings of the 19th World Textile Conference-Autex.

[30] Pooley M.A., Anderson D.M., Beckham H.W., Brennan J.F. (2016). Engineered emissivity of textile fabrics by the inclusion of ceramic particles. Opt. Express.

[31] Anderson D.M., Fessler J.R., Pooley M.A., Seidel S., Hamblin M.R., Beckham H.W., Brennan J.F. (2017). Infrared radiative properties and thermal modeling of ceramic-embedded textile fabrics. Biomed. Opt. Express.

[32] Park C.H., Shim M.H., Shim H.S. (2006). Far IR emission and thermal properties of ceramics coated fabrics by IR thermography. Key Eng. Mater..

[33] Warner S.B. (1995). Fiber Science.

[34] Tao Y., Li T., Yang C., Wang N., Yan F., Li L. (2018). The influence of fiber cross-section on fabric far-infrared properties. Polymers.

[35] Halliday D., Resnick R., Bowen G.H. (1972). Fundamentals of physics. Phys. Today.

[36] Hsu P.-C., Liu C., Song A.Y., Zhang Z., Peng Y., Xie J., Liu K., Wu C.-L., Catrysse P.B., Cai L. (2017). A dual-mode textile for human body radiative heating and cooling. Sci. Adv..

[37] Kamiya H., Gotoh K., Shimada M., Uchikoshi T., Otani Y., Fuji M., Matsusaka S., Matsuyama T., Tatami J., Higashitani K. (2008). Characteristics and behavior of nanoparticles and its dispersion systems. Nanoparticle Technology Handbook.

[38] Hu X., Tian M., Qu L., Zhu S., Han G. (2015). Multifunctional cotton fabrics with graphene/polyurethane coatings with far-infrared emission, electrical conductivity, and ultraviolet-blocking properties. Carbon.

[39] Soares A.R., Pontón P.I., Mancic L., D’Almeida J.R.M., Romao C.P., White M.A., Marinkovic B.A. (2014). Al_2_Mo_3_O_12_/polyethylene composites with reduced coefficient of thermal expansion. J. Mater. Sci..

[40] Salaün F., Vroman I., Bedek G., Lewandowski M. (2008). Effects of microparticles on isotactic polypropylene: Thermomechanical and thermal properties. J. Polym. Sci. Part B Polym. Phys..

[41] Abbas A., Zhao Y., Wang X., Lin T. (2013). Cooling effect of MWCNT-containing composite coatings on cotton fabrics. J. Text. Inst..

